# Novel insights on GM1 and Parkinson's disease: A critical review

**DOI:** 10.1007/s10719-021-10019-7

**Published:** 2022-01-22

**Authors:** Maria Fazzari, Erika Di Biase, Giulia Lunghi, Laura Mauri, Elena Chiricozzi, Sandro Sonnino

**Affiliations:** Department of Medical Biotechnology and Translational Medicine, University of Milan, Milan, Italy

**Keywords:** GM1 ganglioside, GM1 oligosaccharide, Parkinson's disease, Neuronale receptors

## Abstract

GM1 is a crucial component of neuronal membrane residing both in the soma and nerve terminals. As reported in Parkinson’s disease patients, the reduction of GM1 determines the failure of fundamental functional processes leading to cumulative cell distress up to neuron death. This review reports on the role of GM1 in the pathogenesis of the disease, illustrating the current data available but also hypotheses on the additional mechanisms in which GM1 could be involved and which require further study. In the manuscript we discuss these points trying to explain the role of diminished content of brain GM1, particularly in the nigro-striatal system, in Parkinson’s disease etiology and progression.

## GM1 ganglioside

**Fig. 1:**
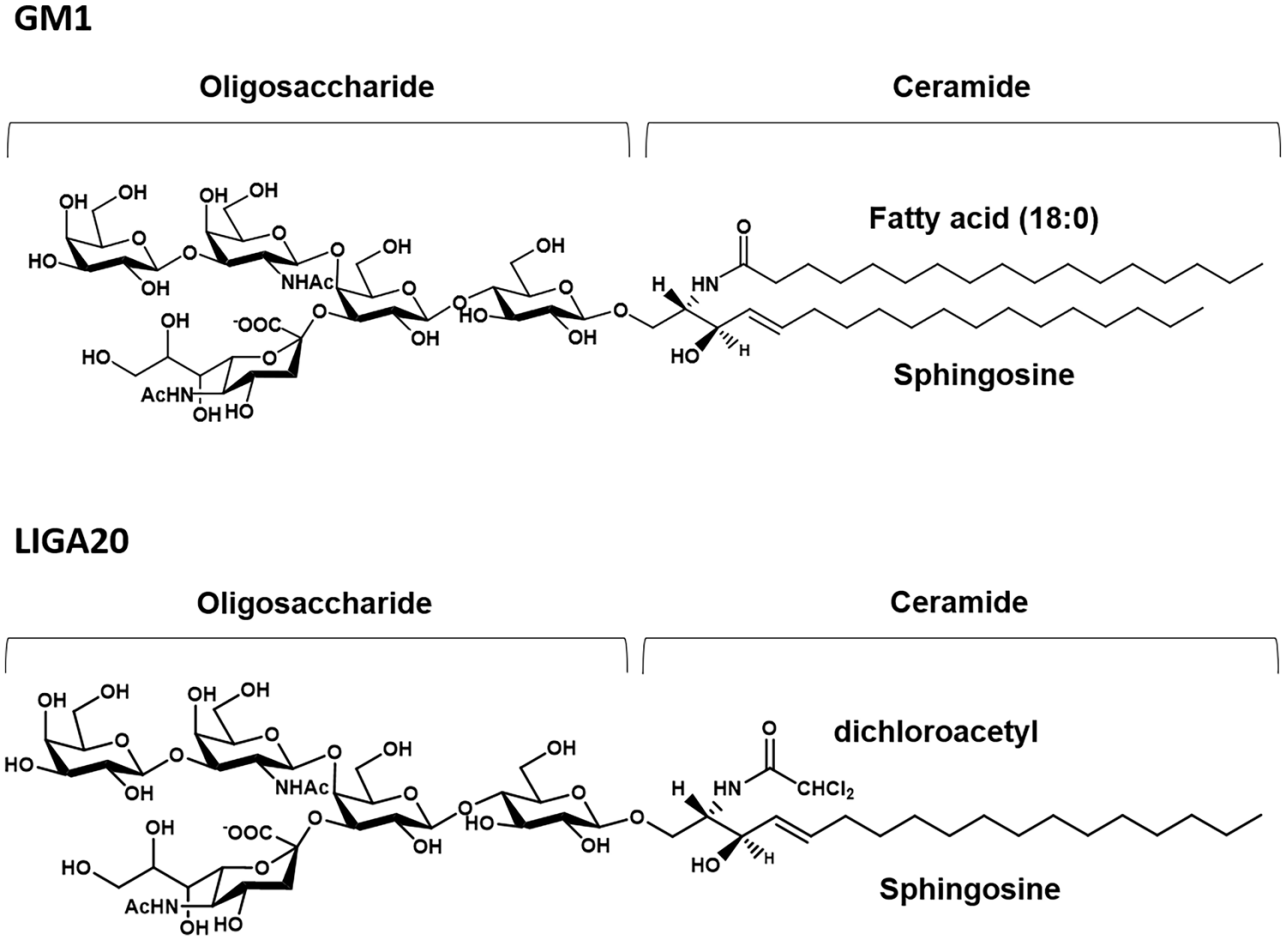
Structure of ganglioside GM1, II3Neu5AcGg4Cer and of its LIGA20 analogue. LIGA20 has a greater ability to cross BBB than GM1 and showed superior neuroprotective efficacy in PD animal models

The ganglioside GM1 (Fig. 1) is an amphiphilic compound displaying a carbohydrate head group with hydrophilicity well balanced by the hydrophobicity of the lipid moiety, the ceramide, inserted into the outer layer of the membrane. Due to intra-residual interactions between the sialic acid lateral chain and the *N*-acetylgalactosamine, the trisaccharide β-GalNAc-(1–4)-[α-Neu5Ac-(2–3)]-β-Gal of GM1 oligosaccharide acts as a single rigid unit conferring a limited dynamic to the moiety [1, 2]. Some mobility is associated to the β-Gal-(1–3)-β-GalNAc- terminal linkage that is available in two couple of torsional angles, this allowing multiple conformers and several interactions with the other membrane components or extracellular ligands [3].

GM1 belongs to the “a” series gangliosides and its biosynthesis occurs in the Golgi apparatus [4]. In addition, a portion of membrane GM1 can be synthesized from complex gangliosides, particularly from GD1a, by the membrane bound sialidase Neu3 that works also in a *trans* fashion on substrates belonging to neighboring cells [5, 6].

GM1 is a component of the neuronal membranes [7], being particularly abundant in the central nervous system, where it covers 10–15% of the total ganglioside content (about 0.9 mg sialic acid lipid bound/g of fresh brain) [8, 9]. GM1 and the more complex gangliosides are enriched in the pre- and postsynaptic membranes of the synaptic terminals [10, 11]. A minor quantity of GM1 is available on the surface of neuronal body. In addition, at lower concentration GM1 can also be found in the non-nervous system tissues [3].

As the other gangliosides, a small amount is present in the cell cytosol complexed with proteins and corresponds to about 3% of the total [12]. We recall that monomers of GM1 are available is solution only at very low concentration not over 10^–9^ M. Over this concentration, GM1 monomers aggregate forming quite spherical micelles, suggesting that stable complexes of monomers with proteins are rapidly formed [13–15].

The peculiarity of the ganglioside structure allows to establish multiple interactions involving: *i*) the hydroxyl groups of the carbohydrate portion that can make hydrogen bonds with other neighboring molecules, *ii*) the amide and hydroxyl groups of the ceramide inserted into the membrane that act as both donors and acceptors of hydrogen bonds and allow lateral interaction with other lipid molecules [16, 17]. These physico-chemical properties of gangliosides provide the driving force for lateral membrane segregation and the formation of rigid floating domains enriched in sphingolipids, cholesterol and palmitoylphosphatidylcholine, named *lipid rafts* [16, 17].

The ability of the cholera toxin to recognize and selectively bind GM1 is used as a common experimental method for the identification of lipid rafts in cells and tissues [18–20].

Among all gangliosides, GM1 is surely that most studied for its bioactive potential. Starting from the beginning of the’80, Scientists rapidly developed studies on GM1, attracted by its neurotrophic and neuroprotective properties [2, 3, 21]. This was facilitated by the large availability of gangliosides that occurred when drugs named *Cronassial* (containing bovine brain ganglioside mixture) and *Sygen* (containing highly purified GM1) were brought to market in 1973 and 1985, respectively, for therapy of peripheral neuropathies [22, 23]. In those years, several clinical trials of different extension, comprising from a few to thousands of patients affected by neurodegenerative diseases, cerebral and spinal injuries were also carried out [24–33].

In the context of Parkinson's disease (PD), the neuroprotective potential of GM1 emerged since the late 1980s studies on mouse and primate disease models exposed to 1-methyl-4-phenyl-1,2,3,6-tetrahydropyridine (MPTP), a neurotoxin that affects the dopaminergic system causing a bioenergetic deficit [34–39]. More recently, the GM1 therapeutic potential has also been confirmed in rats overexpressing the human A53T mutant α-synuclein (αS) via adeno-associated viral vector [40].

The promising preclinical studies supported the development of clinical trials where GM1 replacement therapy demonstrated a modest but significant efficacy improving motor and cognitive outcomes of treated PD patients [41–43]. However, the results lacked strong statistical power to obtain FDA approval as a drug for treating PD, probably due to the small cohort of patients enrolled in these trials [41–43]. Additionally, the GM1 limited bioavailability and blood brain barrier penetrance when systemically administered further hampered its clinical development. In this context, the recent discovery that the oligosaccharide of GM1 represents the bioactive portion provides a new promising drug candidate for PD patients. Thanks to its capability of easily crossing the blood brain barrier, the GM1 oligosaccharide alone is able to perfectly replicate the neurotrophic and neuroprotective properties of the entire GM1 both *in vitro* and *in vivo* [44–51].

## Biosynthesis of GM1 and PD

**Fig. 2:**
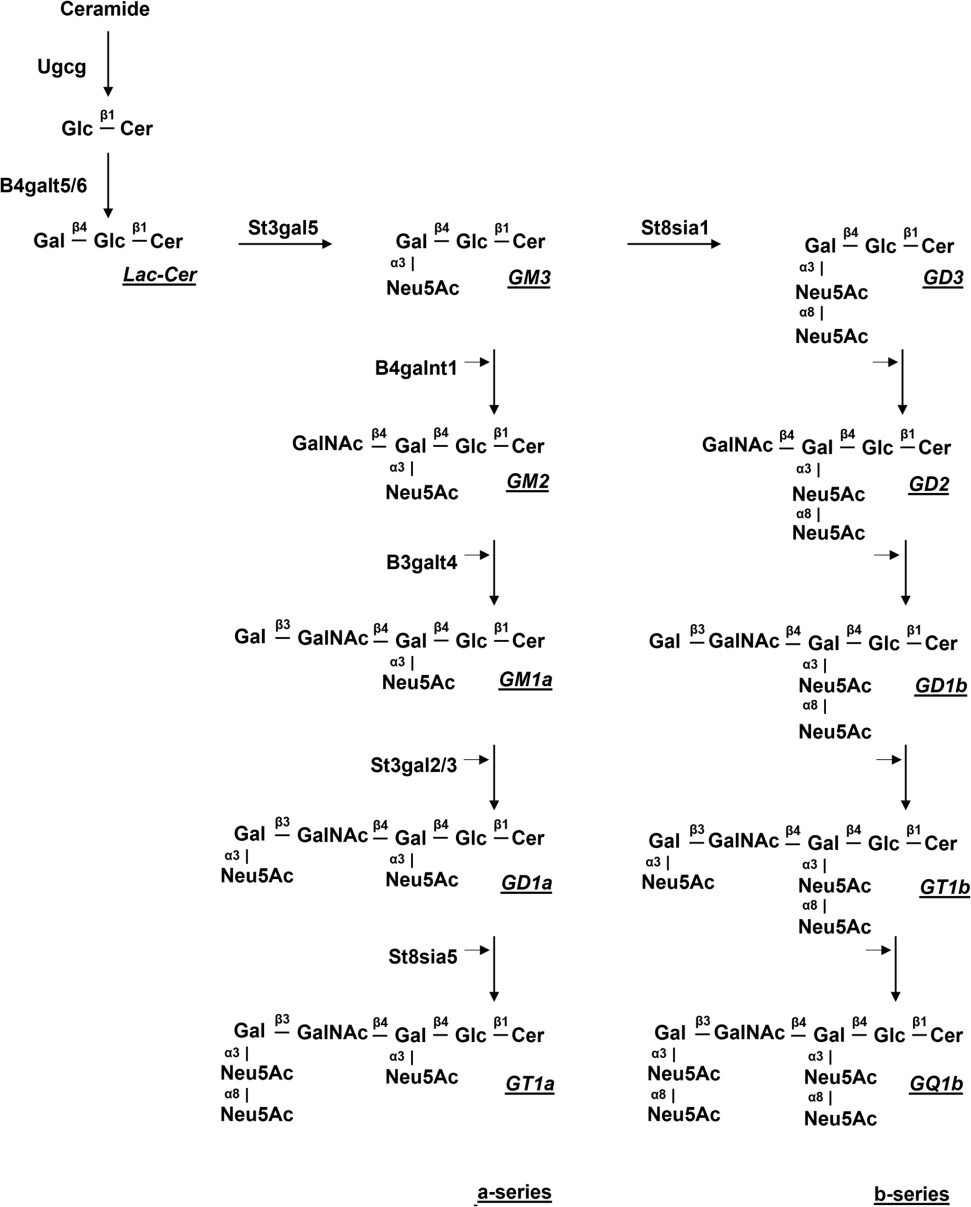
Scheme of a-series and b-series gangliosides biosynthetic pathway. A- and b-series are classified according to the number of sialic acid residues attached to lactosylceramide (Lac-Cer), respectively 1 and 2 residues. The precursor of a series of gangliosides is GM3, directly synthesized from Lac-Cer by the action of the enzyme GM3 synthase (St3gal5). GM3 is converted to GD3 via GD3 synthase (St8sia1) serving as a precursor for the b series. The elongation of the precursors is performed by the sequential action of the following enzymes: GM2/GD2 synthase (B4galnt1), GM1a/GD1b synthase (B3galt4), GD1a/GT1b synthase (St3gal2/3), GT1a/GQ1b synthase (St8sia5). Cer, ceramide

GM1 is the final product of the glycosyltransferase-dependent sequential synthetic process CerGlcCerLacCerGM3GM2GM1, following the scheme reported in Fig. 2. The reduction or the lack of expression of any glycosyltransferase involved in each step limits the availability of GM1 as well as of any following complex ganglioside.

During neuronal differentiation, maturation and synaptogenesis, sialic acid containing glycosphingolipids undergo dramatic changes with the shift from simple gangliosides (GM3, GD3) to complex ones (GM1, GD1a, GD1b and GT1b) [49]. A detailed analysis of ganglioside content in human brains from subjects of different ages revealed that during aging a physiological reduction of GM1 and GD1a occurs and that their content varies significantly among individuals of the same age [52]. Starting from this observation, it was proposed that, in individuals owning a level of a-series gangliosides at the limit of the normal range, the decrease of GM1 and GD1a below the critical threshold value necessary for maintaining crucial neuronal functions [21] can trigger neuropathological dysfunctions leading finally to PD onset [2, 53]. Accordingly, PD patients display a decreased expression of *B4galnt1, B3galt4* and *St3gal2* genes*,* key players for the synthesis of GM1 and more complex gangliosides belonging to b-series [53, 54]. As a consequence, the levels of complex gangliosides, including GM1, GD1a, GD1b and GT1b, are reduced in the *substantia nigra* (SN) [55], in the occipital cortex [56] and in peripheral tissues of PD patients [53]. The aberrant ganglioside synthesis and the consequent depletion of complex gangliosides, especially of a-series, implies an altered membrane composition leading to the vulnerability of dopaminergic neurons. In this view, the dysfunction of ganglioside synthesis can be considered as a causative event underlying the onset of PD, suggesting that a systemic GM1 depletion may be a risk factor in PD.

The direct association between GM1 depletion and Parkinsonian degeneration clearly emerged in the mouse model carrying a loss of function deletion in *B4galnt1* gene [57]. This gene codifies for b-1,4-N-acetylgalactosaminyltransferase 1, the enzyme catalyzing the transfer of N-acetylgalactosamine onto GM3 and GD3 that gives rise to GM2 and GD2 which in turn are metabolized to GM1 and GD1b [58]. Specifically, Wu and coworkers demonstrated that the disruption of *B4galnt1* gene in mice determines the development of typical PD features both in central and in peripheral tissues: motor impairment, loss of dopaminergic neurons from SN, αS aggregation, alterations of gastrointestinal, sympathetic cardiac, and cerebral cognitive systems [56, 57, 59, 60]. Of interest, the heterozygous disruption of the *B4galnt1* gene causing a partial depletion of GM1 comparable to those found in PD patients showed a late-onset but indistinguishable pattern of neurodegeneration if compared to homozygous mice [56, 59]. Remarkably, the administration of exogenous GM1 to *B4galnt1*-defective mice was able to recover both motor and non-motor PD symptomatology, suggesting that GM1 enters the brain even though in limited amounts. In accordance, the treatment resulted more therapeutically effective when mice were injected with a GM1 membrane-permeable analogue (LIGA20) containing the dichloroacetyl group instead of the stearic acid (Fig. 1) [59]. Similarly, the administration of GM1 oligosaccharide portion, owning a high capability to cross blood brain barrier [50], fully recovered both biochemical and behavioral Parkinsonian symptoms of *B4galnt1*^±^ mice [47].

## The GM1-protein interactions

It has been almost 50 years since it was reported that gangliosides interact with proteins and that this is necessary for regulating their activities during physiological processes. Interactions are largely due to the capability of their carbohydrates to form hydrogen bonds, but, in some cases, they are driven by the membrane organization resulting from lipid moiety around the protein and by the hydrophobic forces. The role of GM3 [61], GM1 [45, 62], GD1b [63, 64], GT1b [65] and GQ1b [66] in regulating, positively or negatively, protein processes has been studied in detail. On the other hand, it is necessary to recall that also many neutral glycosphingolipids, with their oligosaccharide chains, interact with microorganism proteins and this is instrumental for bacteria and virus to invade our cells [67, 68].

The first information on the GM1-protein interactions dates back, probably, to the discovery that GM1 is the receptor for the cholera toxin [18–20] and it is involved in recruiting the toxin into the cells. In the following years, the interaction of GM1 with the serum proteins has been studied in detail [69, 70]. GM1 micelles incorporate serum albumin in a ratio of 1:1 (1 micelle:1 protein). The complex slowly and irreversibly forms a big dimeric aggregate. The interaction between GM1 micelles and albumin is mostly hydrophobic. At GM1 submicellar concentrations (lower than 10^−9^ M) the binding of ganglioside monomers to albumin also occurs [70]. This process is due, however, to a non-specific, reversible adhesion of GM1 molecules on the albumin surface with no apparent perturbation of the albumin structure.

**Fig. 3:**
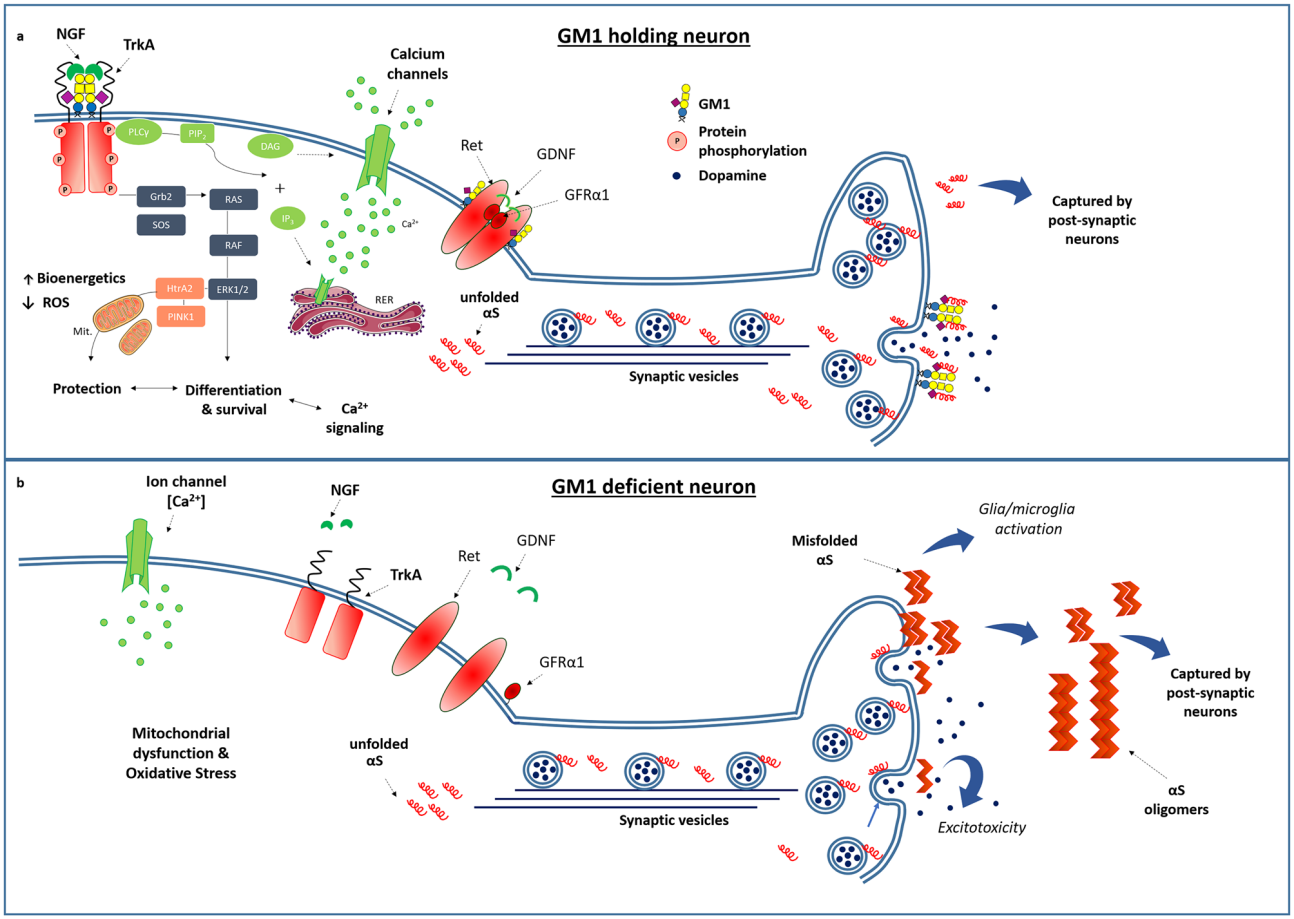
Schematic representation of some neuronal processes requiring GM1. **a** On the top of the image is represented a healthy neuron with normal GM1 levels sustaining the fundamental neuronal signalings and the correct α-synuclein (αS) folding. In particular, GM1 interacts with neurotrophins’ receptors and ion channels mediating neurotrophic and neuroprotective signalings. GM1 is represented also interacting with αS, preventing its pathogenic aggregation. **b** On the bottom, a neuron with GM1 deficiency showing the loss of all the important neurotrophic signals and αS aggregation and accumulation outside the synapsis. In this aggregated form the αS moves towards post-synaptic and other cells causing neuronal distress and the activation of the inflammatory/immune response. Modified from Chiricozzi *et al*. [105]

The importance of GM1 molecule lies in the fact that besides playing a structural role for the formation and maintenance of lipid rafts, it also has a bioactive role. On the cell membrane, GM1 contacts both sensing molecules such as growth factors and neurotrophins and their respective receptors, actively participating in the modulation of transmembrane signaling [2, 21]. In the neurodegenerative and PD context, two main signalings have been reported to be particularly influenced by GM1: the NGF-TrkA and the GDNF-GFRa1-Ret axes. Both signalings support neuronal survival, metabolic activity, plasticity and neuronal transmission (Fig. 3). In the next paragraphs we will deepen the way in which GM1 intervenes in these axes.

Furthermore, the pathognomonic sign of PD is the presence of fibrils and accumulation of the αS protein. Although its function is still unclear today, the relationship between the protein and GM1 has recently been reported [53]. In the section below we will further explore the pathophysiological implications of this relationship.

### The TrkA-GM1 receptor

**Fig. 4:**
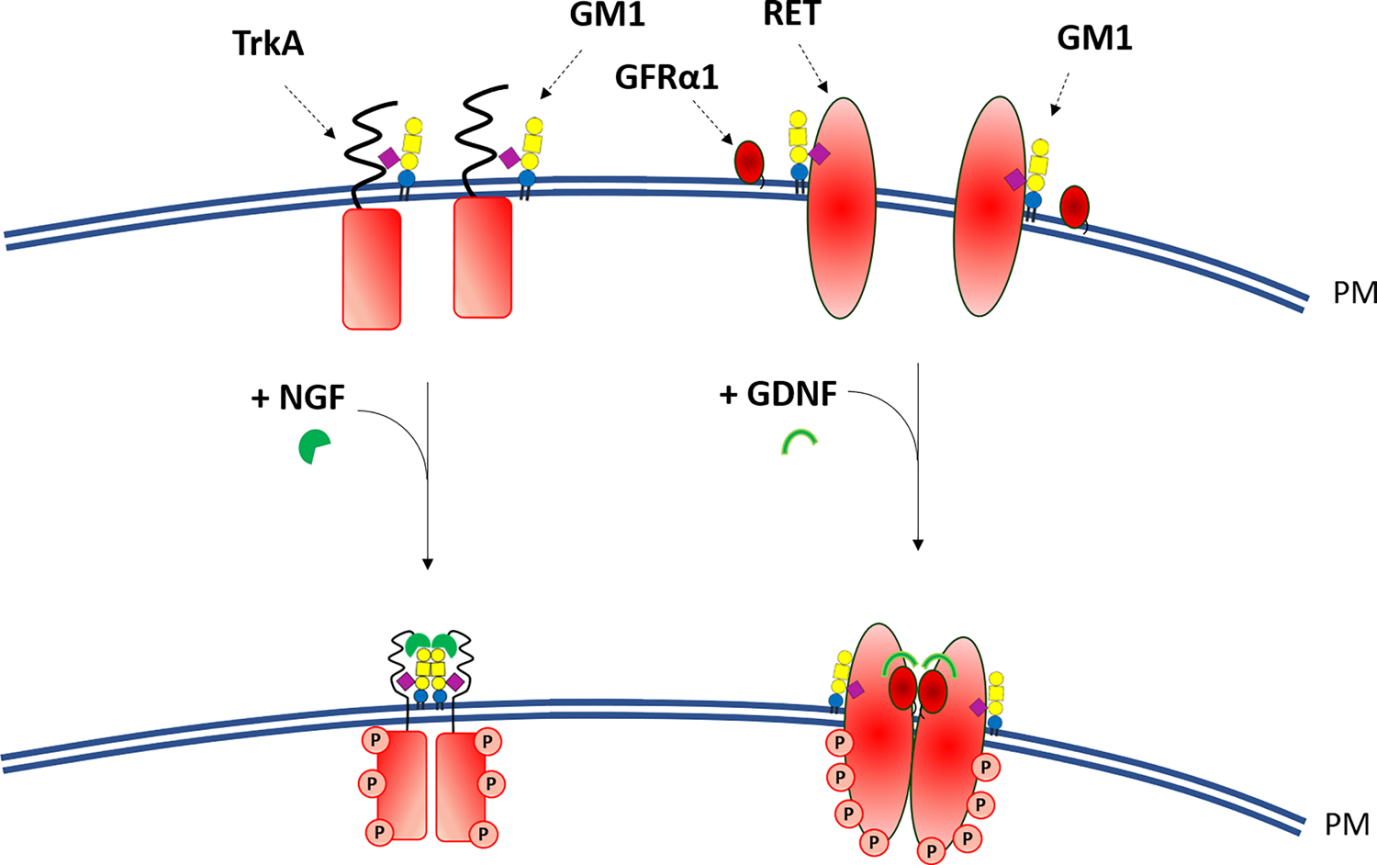
Representation of GM1 interaction with TrkA and Ret receptors at the plasma membrane level. GM1 interacts with TrkA, stabilizing the TrkA-NGF complex, allowing the TrkA autophosphorylation and activation. Adequate levels of GM1 are also necessary to maintain the Ret signaling, stabilizing the tripartite complex GDNF-GFRα-Ret

Neurotrophic factors are key molecules for neuronal differentiation as well as survival of mature neurons. Among them, nerve growth factor (NGF) specifically binds the TrkA receptor at plasma membrane which activation requires the interaction with GM1 oligosaccharide in the extracellular space, thus functioning as a three-component complex TrkA-NGF-GM1 (Fig. 4) [44, 45, 62, 71, 72].

In the presence of NGF, neuroblastoma Neuro2a and pheochromocytoma PC12 cells slowly differentiate in culture, whereas, as expected, the main process occurring is their replication.

Although these cells express the TrkA receptor, they have a low GM1 content and do not synthetize NGF, that can however be provided by the cell culture medium [44, 62]. Interestingly, the addition of exogenous GM1 to cells growth in NGF-containing culture medium arrests the cell replication and rapidly activates the cell differentiation with the formation of neurites [44, 62, 72]. This process is mediated by the activation via autophosphorylation of membrane TrkA receptor triggering the ERK1/2 downstream pathway activation [44, 62, 72]. By administering GM1 oligosaccharide to Neuro2a cells, the potentiation of TrkA phosphorylation is followed by PLCγ and PKC activation with the opening of calcium channels on the plasma membrane and on intracellular storages [51]. Additionally, the GM1 oligosaccharide-induced neurite sprouting was abolished by administration of calcium chelators meaning that the calcium increase is crucial for this neurodifferentiative process [51].

Several detailed studies showed that the GM1 added to cell culture medium becomes a component of the plasma membrane in a time and concentration dependent manner, increasing the original GM1 content of the cell [73–76]. In all studied cell models, a portion of the exogenously administered GM1 is taken up by the cells and associates with the plasma membrane lipid rafts, apparently indistinguishable from the endogenous GM1, thus entering in the basal cell ganglioside metabolic process [73–76]. Repression of the cell synthesis of GM1 by molecular biology procedures in presence of NGF do not induce the differentiation process, suggesting that NGF is not capable of activating the TrkA receptor in the absence of GM1 or at low GM1 cell content [77].

The first study assessing the GM1 direct interaction with TrkA was performed using the low GM1 expressing-PC12 cells [62]. The GM1 administered to the cells could be immuno-precipitated with an anti-TrkA antibody as TrkA-GM1 complex. The complex was separated by polyacrylamide gel electrophoresis (PAGE) and the presence of GM1 identified by cholera toxin [62]. In Neuro2a cells the interaction between TrkA and GM1 was studied by using a photoactivable and tritium-labeled GM1 ganglioside. The administration of this GM1 derivative to cells, followed by illumination, lead to the formation of a complex of covalently linked GM1 and TrkA. The complex being radioactive could be identified by radioimaging after PAGE separation. The use of a GM1 containing the photoactivable group at the end of ceramide moiety or at the external galactose, and of the GM1 oligosaccharide containing the photoactivable group at the C1 of the glucose residue allowed to establish that the interaction of GM1 with the TrkA involves the GM1 oligosaccharide and not the GM1 ceramide [45]. This means that the ceramide of GM1 is not that close to the intramembrane portion of TrkA, while the distance between the oligosaccharide and the extracellular portion of TrkA is short enough to allow the chemical reaction between the two molecules after UV illumination [45].

The GM1-TrkA-NGF complex was studied in detail by molecular docking analyses since the crystallographic structure of the extracellular segment of human TrkA in complex with NGF is currently available and the conformation of the GM1 oligosaccharide as well. This allowed to establish that the addition of GM1 to the TrkA–NGF complex leads to a very stable tri-component complex showing a gain of stability corresponding to about -7 kcal/mol [44].

Comparable results describing the GM1 role on the activity of the TrkA receptor were obtained using mouse primary neurons [78]. At the first h in culture, these cells have very low sialic acid-bound lipids and hardly detectable GM1 [79]. The synthesis of gangliosides proceeds exponentially with the days in culture, parallel to the increase of expression of specific ganglioside glycosyltransferases [79]. The content of gangliosides reaches a plateau in 6 days of culture following the physiological neuronal maturation process. Interestingly, the artificial increase of endogenous GM1 content by administering the ganglioside to the cells during plating determined the acceleration of the neuronal maturation, as indicated by the faster formation of neuronal clusters as well as by the more complex neuronal network with respect to controls [80]. Similarly, the administration of GM1 oligosaccharide to the NGF-expressing cerebellar granule neurons led to enhanced neuronal clustering, arborization and networking together with increased levels of phosphorylation FAK and Src proteins known to be key regulators of neuronal motility [49]. Additionally, GM1 oligosaccharide induced an increased expression of complex ganglioside and specific neuronal markers, suggesting an improved maturation respect to controls [49]. At molecular level, this process was accompanied by the activation of TrkA at cell surface and the following MAPK signaling cascade [49].

All these results lead to the conclusion that NGF is the switch that allow the TrkA signaling cascade but that without GM1 this molecular event cannot proceed. A specific TrkA-GM1 ratio seems to be required, but this probably does not correspond to the 1:1 molar ratio inside the membranes, being the TrkA amount much lower than GM1 even when the GM1 content is very low. This latter point deserves some considerations. GM1 is mainly a component of membrane lipid rafts but TrkA does not belong to these domains in PC12 and Neuro2a cells [45, 81]. The addition of NGF to PC12 cells is sufficient to translocate it into lipid rafts [81], whereas the activation of TrkA by GM1 treatment in Neuro2a cells is not accompanied by the translocation of the receptor into rafts domains [45]. The data available on the crystal structure of the extracellular portion of TrkA suggests that this portion has enough flexibility to lie down on the cell surface finding the GM1 oligosaccharide, this occurring when the GM1-contaning lipid raft is not too far from the TrkA present in its environment. Of course, we cannot exclude that the GM1 interacting with TrkA belongs to that quantity external to the lipid rafts representing the 30–35% of GM1 [3, 82]. In neuronal primary cells, the process seems to be more unclear due to their specific morphology. Indeed, the majority of GM1 is associated to the membranes of nerve endings, while only a minor quantity of the ganglioside, residing inside or outside the lipid rafts, can be found in the neuronal body where TrkA receptor is enriched [3, 82].

In conclusion, the information available supports that GM1 is necessary for the activity of TrkA and that NGF can modulate TrkA function only in presence of a correct amount of GM1 on the cell surface and by interacting with it. This sustains the idea that the true receptor for NGF is the complex TrkA-GM1. However, additional studies are necessary to correctly understand how these membrane events occur. Is the TrkA-GM1 complex firstly available to bind NGF, which then acts to dimerize the TrkA-GM1 complex? Or does the NGF first facilitate TrkA dimerization forming the double-dimer TrkA-NGF-NGF-TrkA complex which is then stabilized by binding to GM1?

### The Ret-GM1-GFRα1 receptor

Ret is a transmembrane receptor with tyrosine kinase activity that, upon dimerization and autophosphorylation, activates the intracellular signaling regulating a myriad of cellular functions: cell survival, differentiation, proliferation, migration, chemotaxis, ureteric bud branching, neurite outgrowth and synaptic plasticity [83]. These Ret functions are regulated by the interaction with the GDNF family ligands (GFLs) to which various neurotrophic factors belong. However, the binding affinity between Ret and GFLs is extremely low and would not occur per se. In fact, the intervention of other molecules, named GDNF-family receptors alpha (GFRα) is required, which act as co-receptors stabilizing the tripartite complex GFL-GFRα-Ret. GFRαs are GPI-anchored proteins of which 4 members are known (GFRα1-4), each specific to a single GFL.

GDNF is a homodimeric protein [84] indispensable for the maintenance and survival of dopaminergic neurons [85]. GDNF is recognized by GFRα1 and their binding allows Ret association in a multiprotein complex with a GDNF_1_(GFRα1)_2_(Ret)_2_ stoichiometry [86] recently confirmed by Cryo-EM [87]. GFRα1 localizes to the lipid rafts and upon interaction with GDNF it associates with GM1, thus recruiting Ret to the rafts domains and promoting its autophosphorylation (Fig. 4) [88, 89]. Only in this way an adequate activation of the raft-associated downstream signaling molecules such as Src kinase is achieved [88]. A major failure of GDNF signaling occurs in PD [56, 58, 90] and seems to be a promising therapeutic target for neuroprotective and regenerative interventions in PD patients [90]. Indeed, PD patients present reduced levels of GDNF and phosphorylated Ret within the SN and striatum [56, 85]. As stated above, suboptimal levels of GM1 have also been reported in the SN of PD patients [59] with a linear correlation between the levels of GM1 and the degree of Ret phosphorylation in the SN dopaminergic neurons of PD patients [56]. In this regard, the importance of GM1 is evident from the *B4galnt1*^±^ mouse model, deficient for all gangliosides including GM1. This mouse strain develops parkinsonism in old age with a strong deficit of GDNF signaling which is rescued following the administration of the GM1 analog, LIGA20 [56, 59]. In fact, LIGA20 infusion restores the adequate level of Ret phosphorylation and the downstream transducers (Src and MAPK), allowing a recovery of both the molecular signaling and phenotypic pathological features. Adequate levels of GM1 are therefore necessary to maintain GDNF / Ret signaling, despite its minority percentage in neuronal lipid rafts when compared with GD1a, GD1b and GT1b. However, none of the latter gangliosides except GM1 is co-immunoprecipitated with phosphorylated Ret and GFRα1 in the SN of wt and *B4galnt1*^±^ mice. Which are the sites of interaction between GDNF-GFRa1-Ret and GM1 and which is the role played by the specific sequence of the GM1-oligosaccharide are still open questions to date.

### α-Synuclein and GM1─protein interactions

Pathognomonic sign of PD is the presence of fibrils accumulation of the αS protein, which forms intracellular and intercellular inclusions known as "Lewy bodies" by capturing inside dozens of other proteins and lipidic species [91]. αS is a small protein (140 aa) classically defined as intrinsically disordered [92, 93], later found *in vivo* as a tetramer enriched in α-helices domains due to the presence to the N-terminal α-acetyl group [94]. For reasons not yet completely understood, this protein can undergo conformational switching towards parallel β sheets enrichment that favors its aggregation first in oligomers and then in progressively longer fibrils, causing cellular distress, alteration of neuronal function and immune response activation [95]. Despite numerous studies on αS, its function is still debated. It has a cytoplasmic localization with enrichment in the axon terminals [96], where it plays a role in mediating the processes of fusion and invagination of the synaptic vesicles. In fact, besides the interaction with SNARE protein [96], αS interacts with cellular lipids, mostly acidic ones [97, 98]. Studies using synthetic membranes have recently shown that αS has a negligible affinity for the outer plasma membrane which increases considerably when enriched in gangliosides such as GM1 [99]. However, αS has a great affinity for both the inner plasma membrane (IPM) and the membrane of the presynaptic vesicles (SVs), acting as a double anchor that binds the IPM with one arm and the SV with the other one, thus docking the SV to the membrane. The portion by which the αS binds to the IPM also tends to bind the SVs each other, thus acting as a glue for the clustering of SVs in the presynaptic terminal. This is possible because αS is a protein whose conformational dynamism is strictly dependent on the presence and composition of the lipids of the membranous structures to which it is associated. With similar biophysical techniques on synthetic membranes enriched with gangliosides, it was found that GM1 strongly interacts with αS [100, 101]. Furthermore, GM1 stabilizes the non-amyloidogenic α-helix conformation of the protein, reason why GM1 was thought as a neuroprotective molecule, precisely in preventing pathological fibrillation of the αS. This aspect was further investigated *in vivo* where a functional correlation between GM1 and αS was identified: a reduction of GM1 in the membrane of dopaminergic neurons increases the risk of αS and Lewy body aggregates. Surprisingly, replacement therapy with LIGA20 (the permeable analogue of GM1) reduces these aggregates, highlighting a strong relationship between the levels of GM1 and αS accumulation [59, 60]. Specifically, the GM1 dis-aggregative properties seems to be attributable to its oligosaccharide chain: the administration of GM1 oligosaccharide *in vivo* led to a significant reduction of αS accumulation [47]. Further, the GM1 injection in rats overexpressing human mutant αS (A53T) in SN was found to reduce αS aggregation in striatum, displaying neurorestorative effects on nigro-striatal system [40].

A question inevitably arises from the following evaluations. It is established that the primary localization of GM1 and αS is the same, that is at the level of axon terminals membranes, however the two "operate" in diametrically opposite regions: αS works on the cytoplasmic side of the plasma membrane by mediating SVs docking and clustering, while GM1 is located on the outer layer of the membrane. How then is an interaction between the two physiologically made possible? It is known, as previously mentioned, that a small amount of GM1 is also present in the cytoplasm complexed with proteins. It is therefore speculated that this small amount of cytosolic GM1 could be responsible for the stabilizing effects of the αS folding. There are very few studies on cytoplasmic GM1 and associated proteins. Only in one study, by photolabeling and crosslinking techniques, the authors isolated the proteins to which cytoplasmic GM1 was bound in human fibroblasts, identifying a pool of few but specific protein bands with molecular mass ranging from 30 kDa to about 100 kDa [12]. Monomeric αS weights about 14 kDa and appears to be excluded from the range, however fibroblasts express very low levels of αS under physiological conditions. Thus, further studies aimed at demonstrating the putative relationship between cytoplasmic GM1 and αS are still required as well as information about the role that plasma membrane GM1 could play in the physiopathology of αS. Indeed, αS is released into the synaptic extracellular space upon neuronal activity [102], yet the dynamic of the process has not been clarified. During this process the rigid lipid raft domains where GM1 is enriched could be capable to block αS spreading, a possible mechanism by which GM1 could avoid the protein aggregation [103]. To better understand this process it would be necessary to know how αS linked to the external layer of the vesicles is capable to move, by diffusion or through specific channels, to the external layer of synaptic membrane following vesicle fusion [102]. Alternatively, we should consider that during membrane fusion, important membrane rearrangements could occur.

Lipid domains are rigid but very dynamic platform and any change in the content of their components requires a composition rearrangement [104]. We cannot exclude that a reduced synthesis of GM1 and GD1a makes the synaptic lipid raft no more suitable for interaction with αS. At this point, αS would start with aggregation entering in such form into the post-synapsis and neural body, as well as in other brain cells.

Collectively, from the above, it clearly emerges the crucial role of GM1 ganglioside in maintaining the healthy status of nervous system. Although the precise mechanisms involved should be clarified, the GM1 capability to modulate the neurotrophins’ signalings and to avoid αS aggregation clearly highlights its role as regulator of neuronal homeostasis, capable of preventing the neurodegeneration. Thus, therapeutic strategies aimed at elevating GM1 levels are extremely attractive in PD context. However, only a low amount of peripherally administered GM1 can effectively reach the damaged neurons. The development of delivery strategies or the use of more permeable modified-GM1 can be useful. In this regard, the spotlight seems to be on the GM1 oligosaccharide, a brain-permeable small molecule maintaining all beneficial effects of the ganglioside from which it derives, thus demonstrating a strong therapeutic potential.
